# Clustering of Dietary Patterns and Lifestyles Among Spanish Children in the EsNuPI Study †

**DOI:** 10.3390/nu12092536

**Published:** 2020-08-21

**Authors:** Julio Plaza-Díaz, Esther Molina-Montes, María José Soto-Méndez, Casandra Madrigal, Ángela Hernández-Ruiz, Teresa Valero, Federico Lara Villoslada, Rosaura Leis, Emilio Martínez de Victoria, José Manuel Moreno, Rosa M. Ortega, María Dolores Ruiz-López, Gregorio Varela-Moreiras, Ángel Gil

**Affiliations:** 1 Department of Biochemistry and Molecular Biology II, School of Pharmacy, University of Granada, 18071 Granada, Spain; jrplaza@ugr.es; 2 Institute of Nutrition and Food Technology “José Mataix”, Center of Biomedical Research, University of Granada, Avda. del Conocimiento s/n, 18016 Granada, Spain; memolina@ugr.es (E.M.-M.); emiliom@ugr.es (E.M.d.V.); mdruiz@ugr.es (M.D.R.-L.); 3 Instituto de Investigación Biosanitaria IBS.GRANADA, Complejo Hospitalario Universitario de Granada, 18014 Granada, Spain; 4 Department of Nutrition and Food Sciences, Faculty of Pharmacy, University of Granada, 18071 Granada, Spain; casandram@correo.ugr.es; 5 Iberoamerican Nutrition Foundation (FINUT), Armilla, 18016 Granada, Spain; msoto@finut.org (M.J.S.-M.); ahernandez@finut.org (Á.H.-R.); 6 Spanish Nutrition Foundation (FEN), 28010 Madrid, Spain; tvalero@fen.org.es (T.V.); gvarela@ceu.es (G.V.-M.); 7 Instituto de Nutrición Puleva, 18004 Granada, Spain; federico.lara@lactalis.es; 8 Department of Pediatrics, Unit of Pediatric Gastroenterology, Hepatology and Nutrition University Clinical Hospital of Santiago, 15706 Santiago de Compostela, Spain; mariarosaura.leis@usc.es; 9 Instituto de Investigación Sanitaria de Santiago, IDIS, Santiago de Compostela, University Clinical Hospital of Santiago, 15706 Santiago de Compostela, Spain; 10 CIBEROBN (Physiopathology of Obesity and Nutrition), Instituto de Salud Carlos III (ISCIII), 28029 Madrid, Spain; 11 Department of Physiology, University of Granada, 18071 Granada, Spain; 12 Department of Pediatrics, University of Navarra Clinic, 28027 Madrid, Spain; jmorenov@unav.es; 13 Department of Nutrition and Food Sciences, Faculty of Pharmacy, Complutense University, 28040 Madrid, Spain; rortega@ucm.es; 14 Department of Pharmaceutical and Health Sciences, Faculty of Pharmacy, CEU San Pablo University, 28668 Madrid, Spain

**Keywords:** children, cluster analysis, dietary patterns, physical activity, sedentary behavior

## Abstract

Dietary patterns (DPs) are known to be tied to lifestyle behaviors. Understanding DPs and their relationships with lifestyle factors can help to prevent children from engaging in unhealthy dietary practices. We aimed to describe DPs in Spanish children aged 1 to <10 years and to examine their associations with sociodemographic and lifestyle variables. The consumption of toddler and young children milk formulas, enriched and fortified milk within the Spanish pediatric population is increasing, and there is a lack of evidence whether the consumption of this type of milk is causing an impact on nutrient intakes and if they are helping to reach the nutrient recommendations. Within the Nutritional Study in the Spanish Pediatric Population (EsNuPI), we considered two study cohorts and three different age groups in three year-intervals in each of them. The study cohort included 740 children in a representative sample of the urban non-vegan Spanish population and 772 children in a convenience cohort of adapted milk consumers (AMS) (including follow-on formula, toddler’s milk, growing up milk, and fortified and enriched milks) who provided information about sociodemographics, lifestyle, and dietary habits; a food frequency questionnaire was used for the latter. Principal component analysis was performed to identify DPs from 18 food groups. Food groups and sociodemographic/lifestyle variables were combined through a hierarchical cluster algorithm. Three DPs predominated in every age group and study sample: a palatable energy-dense food dietary pattern, and two Mediterranean-like DPs. However, children from the AMS showed a predominant dietary pattern markedly related to the Mediterranean diet, with high consumption of cereals, fruits and vegetables, as well as milk and dairy products. The age of children and certain lifestyle factors, namely level of physical activity, parental education, and household income, correlated closely with the dietary clusters. Thus, the findings provide insight into designing lifestyle interventions that could reverse the appearance of unhealthy DPs in the Spanish child population.

## 1. Introduction

Infancy is a stage characterized by high nutrient requirements and rapid conversion from a primarily milk-based diet (breast milk or infant formula) to a diverse diet with the ingestion of numerous food groups. The first two years of life are critical in individual growth and can disturb long-term health [1]. Later, in childhood, periods of dietary change are apparent between the ages of three and four years, and the ages of seven and nine years [2]. Parental food choice and the family and school environments mainly interact to shape young children’s eating behavior [3], which may have consequences for the incidence of overweight and obesity and derived non-communicable chronic diseases (NCCDs). Poor dietary habits are associated with a higher prevalence of NCCDs and increased mortality in both developing and developed countries [4]. Unhealthy dietary and lifestyle behaviors—such as sedentarism, mainly associated with increased screen-time, and low physical activity time—are established in childhood and tend to track throughout adolescence and into adulthood [5,6,7]. Notably, in Spanish children aged three to five years, dietary and other lifestyle habits appear to be influenced by their parents’ health awareness and other socio-economic characteristics [8]. Therefore, healthy lifestyle behaviors should be established early in childhood.

Most dietary guidelines in many countries promote the consumption of milk and other dairy foods, e.g., fermented milk and cheese, as they provide high-quality protein, calcium, and a wide range of micronutrients [9,10,11]. Emerging evidence shows that the consumption of dairy foods has a neutral or inverse association with adverse cardiometabolic health outcomes, including atherosclerotic cardiovascular disease, type 2 diabetes, and associated risk factors [12,13]. However, the consumption of dairy is decreasing away from the recommended level in many countries, and the potential benefits of milk and dairy products for health are being questioned [12], despite regular consumption of dairy being associated with linear growth and bone mineral content in childhood and adolescence [14]. European children with a healthier lifestyle consume more milk and yogurt, and, in Spain, those who consumed more milk also had better dietary patterns [15,16].

A balanced diet in children covers the needs for all the nutrients. However, in developed countries, i.e., Spain, some nutrients are commonly consumed in excess (e.g., protein), or a percentage of children ingest insufficient amounts of others, primarily iron, calcium, vitamin D, and docosahexaenoic acid (DHA) [17,18]. The use of young child formulas in Spain include follow-on formulas (food intended for use as a liquid part of the weaning diet for the infant from the 6th month on and for young children), toddler’s milk (targeted to young children between 12 and 36 months, typically sold as a line of products, and labelled as stages 1, 2, and 3 depending on target age groups), termed in Spain “growing up milks”, and fortified milks with selected nutrients can help meet the requirements in such situations, but this has been little explored [19,20]. The consumption of young milk formulas (follow-on and toddler’s milk) and enriched and fortified milk within the Spanish pediatric population. They are consumed at around 9225 tons per year and are gaining importance and increased sales. According to the Food Consumption Report in Spain 2018, sales of fortified milk increased by 26.3% compared to 2017, and there is a lack of evidence regarding whether the consumption of this type of milk is causing an impact on nutrient intakes and whether it is helping to reach nutrient recommendations [21].

Food consumption studies traditionally focused on food ingredients and nutrients. However, since diet is multidimensional and complex, other approaches have been developed in recent years, and research has shifted toward approaches focusing on dietary patterns (DPs) [22,23]. Human choices are shaped by individual, societal, and cultural factors [24]. Since the early 1980s, DPs have been used to synthesize multiple related dietary components in combined variables representing key dietary habits and the overall diet in free-living individuals. Interest in DPs is also motivated by the well-known interactive effects of foods that are eaten together and by data dimensionality and multiple testing issues affecting the statistical analysis of many single food groups or nutrients [25].

Principal component analysis (PCA), exploratory factor analysis, or cluster analysis have been employed to empirically derive DPs [26]. These decisions concern input variable format and potential transformation, number of input variables and food grouping schemes, estimation method, and criteria for model selection, including how to choose the number of DPs to retain [27]. Despite clear differences in approaches and interpretations, there is some evidence that underlying eating patterns are revealed by either PCA or cluster analysis [23] and most identified DPs generate valid dietary constructs characterizing the populations under consideration [24].

Several studies used the methods mentioned above to gain insight into the relationships between diet, physical activity, sedentary behavior, and country-specific DPs among children and adolescents [28,29,30,31,32]. For example, seven DPs were detected in 3427 children aged six years in Brazil [33], another study analyzed the food intake of 1130 children aged one to five years from the Czech Republic without identification on DPs [34], and three DPs were identified in 1063 children (six to eight years) from Coimbra, Portugal [4]. In a European study carried out in 9301 children aged two to nine years, three DPs based on a higher frequency of consumption of snacks and fast food (processed), sweet foods and drinks (sweet), and fruits and vegetables (healthy) were found [35]. Other studies showed that Mediterranean diet adherence is directly associated with physical activity (and possibly with diet adequacy) and inversely with sedentary behavior, whereas the results for gender, age, socioeconomic status, and weight status were inconsistent [36].

In Spain, the ANIBES (“Anthropometric data, macronutrients and micronutrients intake, practice of physical activity, socioeconomic data and lifestyles in Spain”) study focused on DPs, physical activity behaviors, sedentary behaviors, and sleep time on weekdays in 207 Spanish children aged 9–12 years and 208 adolescents aged 13–17 years, clustered into two different groups (a low physical activity and poorer diet lifestyle pattern, which included a higher proportion of girls, and a high physical activity, low sedentary behavior, longer sleep duration, healthier diet lifestyle pattern) [37]. Leis et al. [38] assigned 489 children from the GENOBOX (Associations between genetic variants, biomarkers of oxidative stress, inflammation and cardiovascular risk in obese children) study (children in good health and normal weight, overweight, and with obesity, 5–14 years of age) to three clusters. Children from cluster 1 exhibited higher plasma insulin, homeostatic model assessment for insulin resistance (HOMA-IR), and triacylglycerols compared with the other groups [35]. However, relevant information for other age groups (less than five years) in Spain is lacking. For this reason, the Nutritional Study in Spanish Pediatric Population (EsNuPI) study was designed to determine the food consumption, nutrient intake, and physical activity and sedentary behaviors of Spanish children from 1 to <10 years old in urban areas with >50,000 inhabitants distributed across nine geographical areas (according to the Nielsen Spanish criteria) [21].

Given this background, we aimed to (1) determine the DPs in a representative cohort of the Spanish non-vegan pediatric population, living in urban areas (SRS) in three different age groups (group 1 (Gp 1), 1 to <3 years old; group 2 (Gp 2), 3 to <6 years old, and group 3 (Gp 3), 6 to <10 years old) as well as in a convenience cohort of adapted milk consumers (including follow-on formula, toddler’s milk, growing up milk, fortified and enriched milks) of the same age groups (adapted milk consumers cohort, AMS), and (2) examine how sociodemographic and lifestyle factors are clustered within the DPs and among the three age groups.

## 2. Materials and Methods

### 2.1. Study Design and Samples

The data used in this manuscript were obtained as part of the EsNuPI study, which was a prospective, cross-sectional, observational study, conducted from October 2018 to January 2019. The complete design, protocol, and methodology of the EsNuPI study were described in detail elsewhere [21]. Briefly, the EsNuPI study evaluated the dietary and nutrient intake and dietary patterns, as well as physical activity and sedentary behaviors of non-vegan Spanish children, living in urban areas, distributed in nine regions according to Nielsen Spanish areas. Nielsen geographical distribution of the sample for the EsNuPI study: area 1 (9.8%), northeast; area 2 (12.6%), Levant (east); area 3 (15.1%), south; area 4 (7.6%), central; area 5 (11.9%), northwest; area 6 (8.6%), north central; area 7 (9.4%), metropolitan area of Barcelona; area 8 (19.0%), metropolitan area of Madrid; and area 9 (6.0%), Canary Islands [21].

Two subsamples were selected, one SRS and one AMS both from 1 to <10 years old. The AMS regularly consumed follow-on formula, toddler’s milk, fortified or enriched milk depending of the specific children age. All these formulas are usually formulated with a partial replacement of saturated fatty acids (SFAs) from milk fat with unsaturated fatty acids (FAs), from vegetable oils and added with selected nutrients, namely calcium, iron, vitamin D, and docosahexaenoic acid (from marine oil) depending on the specific age of the child. The reasons for choosing the AMS and SRS cohorts are described elsewhere [21].

A total of 1514 children agreed to participate in the study and completed the face-to-face interview. The EsNuPI study was conducted following the Declaration of Helsinki and was approved by the University of Granada ethical committee (No. 659/CEIH/2018) and registered in ClinicalTrials.gov (Unique Protocol ID: FF01/2019).

#### 2.1.1. Dietary Survey and Data Collection

For our study, a food frequency questionnaire (FFQ) was used that was previously modified, adapted, and validated with the portion sizes and food groups usually consumed by the Spanish child population [39]. This FFQ was a quantitative one, and participants answered questions regarding their child’s usual food consumption over the last 12 months, structured by 10 food groups and including 251 different foods including the foods and beverages most commonly consumed by the Spanish population [21,39,40]. Then, these 10 groups were divided into 18 groups due to the wide diversity of food products currently available. The main food groups considered in this study were as follows: “milk and dairy products”, “cereals”, “meat and meat products”, “oils and fats”, “bakery and pastry”, “fruits”, “vegetables”, “sugars and sweets”, “ready to cook” (is industrial pre-cooked foods prepared with the expectation they will be heated/cooked by frying, microwave, oven, stovetop (e.g., frozen pizza, frozen vegetables, etc.) or baking), “other dairy products”, “beverages”, “legumes”, “eggs”, “fish and shellfish”, “appetizers”, “cereal-based baby foods”, “nuts”, and “sauces and condiments” [41].

#### 2.1.2. Physical Activity and Sedentary Behavior Questionnaire

The physical activity and sedentary behaviors questionnaire used for this study was a modification of a questionnaire previously validated in children aged <10 years from Colombia based on a seven-day recall [42]. Some modifications, mostly in commonly used language terms, were made to this questionnaire to adapt it to the needs of the present study.

The questionnaire provided information on the physical activity and sedentary behaviors in children under 10 years of age. Furthermore, we adjusted the questionnaire to the Spanish most common terms of different activities. The detailed protocol has been reported elsewhere [21]. In brief, physical activity was reported indicating all the activities performed by the child in one day (24 h) during the last week (a seven-day record), including hours of sleep and screen time, and was reported separately for weekdays and weekends [21]. This questionnaire included activities that require more effort (cycling, walking, dancing, jumping, etc.) and activities that require little or no effort (reading time/homework, hours spent watching television, time spent using the computer and game consoles or screens, foreign language classes, music or drawing courses). Participants could list other activities that were not included in the other sections. Sleeping and eating habits included the number of hours each child slept per night and duration of naps, as well as the number of hours eating, on average, and they were reported separately for weekdays and weekends.

Finally, the calculations of energy cost were based on the metabolic equivalents (METy) value from the Youth Compendium, a measured or computed basal metabolic rate (BMR), and the duration of each specific activity, as follows:

Energy cost (kcal) = METy × BMR (kcal/min) × duration (minutes), where the BMR for boys and girls was predicted using specific Schofield equations [43,44,45]. Physical activity level (PAL) was calculated using the mean 24 h total energy expenditure (TEE) and BMR in the following equation: PAL = TEE/BMR. Energy expenditure moderate–vigorous physical activity (EE-MVPA) was calculated using the mean 24 h total energy expenditure for PAL ≥1.6.

#### 2.1.3. Anthropometric Data

Weight and size data were reported by parents or caregivers based on the latest child’s health card. The weight and height of the children were evaluated using the World Health Organization (WHO) international growth patterns based on the analysis of the body mass index (BMI)-for-age indicators. For each child, the z-score index was estimated using WHO Anthro and WHO Anthro PLUS software (Version 3.2.2, January 2011).

#### 2.1.4. Covariates

##### Parental Education

The education levels of parents participating children were established following the Spanish educational system. After a preliminary analysis of the distribution of the variable, categories were collapsed and recoded into a 3-point scale as follows: (1) low (less than 10 years of education, primary school or less), (2) medium (12 years of education; higher secondary education), and (3) high (13 years or more of education; higher vocational, college, and university studies) [21].

##### Household income (HI)

Household income (HI) was classified based on occupation (12 categories) according to the National Association of Opinion and Market Research (ANEIMO) criteria, which were adapted to the Spanish context using the World Association for Market, Social and Opinion Research (ESOMAR) criteria. After a preliminary analysis of the distribution of the variable for this analysis, the categories were collapsed and recoded into a 3-point scale as follows: (1) low, (2) mid-low, and (3) high (mid-mid, mid-high and high) HI levels [21].

### 2.2. Data Analysis

Statistical tests were performed using IBM SPSS Statistics for Windows, Version 25.0 (IBM Corp., Armonk, NY, USA), and R version 3.6.1 (R Foundation for Statistical Computing, Vienna, Austria). Descriptive statistics were computed for each variable. All results are expressed as the mean ± standard deviation unless otherwise indicated. *p*-values were obtained from H Kruskal–Wallis tests corrected by the Bonferroni post hoc test when age groups were compared for each sample. The chi-squared test was used to compare percentage differences for parental education and household income variables.

#### 2.2.1. Dietary Patterns

PCA was performed to identify underlying dietary patterns using the average weight consumed (g/day) by each individual from 18 food groups as input variables [39]. Multicollinearity was evaluated by looking at the determinant of the R-matrix Bartlett’s test of sphericity, and the Kaiser–Meyer–Olkin (KMO) measure of sampling adequacy was used to verify the appropriateness of factor analysis. To assess the degree of intercorrelations between variables, we adopted a value >0.60 for the KMO. Factors were orthogonally rotated (the Varimax option) to enhance the difference between loadings, which facilitated interpretability.

Factors were retained based on the following criteria: factor eigenvalue >1.2, identification of a breakpoint in the scree plot, the proportion of variance explained, and factor interpretability [26].

The strength and direction of the associations between patterns and food groups were described through a rotated factor loading matrix. Food groups with factor loadings >0.20 and communality >0.20 were retained in the patterns identified. The factor score for each pattern was constructed by summing observed intakes of the component food items weighted by the factor loading. A high factor score for a given pattern indicated a high consumption of the foods constituting that food factor, and a low score indicated a low intake of those foods. Radar charts were used to display multivariate data in the form of a two-dimensional chart of 18 food groups (input variables) represented on axes starting from the same point.

#### 2.2.2. Lifestyle Patterns with Clustering Analysis

A two-step cluster analysis procedure was used as an exploratory tool to reveal natural clusters within the dataset that would otherwise not be apparent and automatically determine the “best” number of clusters using SPSS v.25 (IBM, Chicago, IL, USA). Thereafter, using R v.3.6.3 package, unsupervised hierarchical clustering analysis was applied on the FFQ and lifestyle variables (anthropometric measure, parental education, HI), to build clusters of subjects with similar characteristics (R package pheatmap). The distance matrix was defined by Euclidean distances, and Ward’s method was used as linkage criteria to group the clusters. More specifically, based on the distance matrix, the clustering algorithm identified the closest observations (i.e., subjects with similar dietary and lifestyle behaviors, in rows) and iteratively merged them within the same cluster until all clusters were merged together. This clustering algorithm was applied separately within each age group. Three clusters were retained among subjects and lifestyle/dietary variables, which were assumed to be the optimum number of clusters (2 to 5 clusters were tested) based on the silhouette method (R package Nbclust). The agglomerative coefficient, calculated by the Agnes function in R package cluster, was always higher than 0.85.

## 3. Results

### 3.1. Description of the Sample

A total of 1512 children of the EsNuPI study sample whose parents or caregivers agreed to participate and completed the FFQ (49.9% girls and 50.1% boys) were analyzed. The total SRS represented 48.9%, and the AMS 51.1%. The characteristics of both study samples are provided in Table 1. We did not exclude any participants based on potential misreporting of energy intake as energy intake for both the SRS and AMS cohorts were not significantly different from those of plausible reporters [21].

The food consumption divided according to the 18 food groups considered in the present study is shown in Table 2. In SRS, the use of milk and dairy products was significantly higher in children from age 1 to <3 years compared with children from 3 to <6 and 6 to <10 years, whereas the consumption of meat and meat products, oils and fats, other dairy products, sugars and sweets, eggs, beverages, and appetizers was significantly lower. Consumption of bakery and pastry, vegetables, ready to cook, fish and shellfish, cereal-based baby foods, sauces and condiments, and nut consumption was significantly different in the three age groups (Table 2). While bakery and pastry, ready to cook, fish and shellfish, sauces and condiments, and nut consumption increased, the use of vegetables and cereal-based baby foods decreased. In AMS, children from 3 to <6 and 6 to <10 years had an analogous consumption of milk and dairy products, cereals, meat and meat products, fruits, vegetables, and eggs in comparison with children aged 1 to <3 years. In this sample, milk and dairy products, cereals, fruits, and vegetable consumption values were higher than those in children aged 3 to <6 and 6 to <10 years. Meat and meat products and egg consumption values were lower in children aged 1 to <3 years when compared to those of children aged 3 to <6 and 6 to <10 years. Bakery and pastry, other dairy products, sugars and sweets, ready to cook, fish and shellfish, beverages, cereal-based baby foods, appetizers, sauces and condiments, and nut consumption values were significantly different across age groups (Table 2). Bakery and pastry, other dairy products, sugars and sweets, ready to cook, fish and shellfish, beverages, appetizers, sauces and condiments and nut consumption increased with age, whereas cereal-based baby foods consumption decreased according to age (Table 2).

Comparing the total cohorts (SRS vs. AMS), we found that consumption of meat and meat products, bakery and pastry, other dairy products, sugars and sweets, ready to cook, eggs, fish and shellfish, beverages, appetizers, and nuts group foods was significantly higher in SRS, whereas milk and dairy products, vegetables, cereal-based baby foods, and sauces and condiment consumption was significantly higher in AMS (Table 2).

Between-group ages, we found that children aged 1 to <3 years consumed more milk and dairy products and cereal-based baby foods in AMS than in SRS. In contrast, sugar and sweet consumption was significantly higher in SRS than in AMS. Children aged 3 to <6 years consumed more beverages in SRS than in AMS. In children aged 6 to <10 years, we observed a higher consumption of oils and fats, appetizers, and sauces and condiments in AMS than in SRS (Table 2).

### 3.2. Dietary Patterns

**Figure 1:**
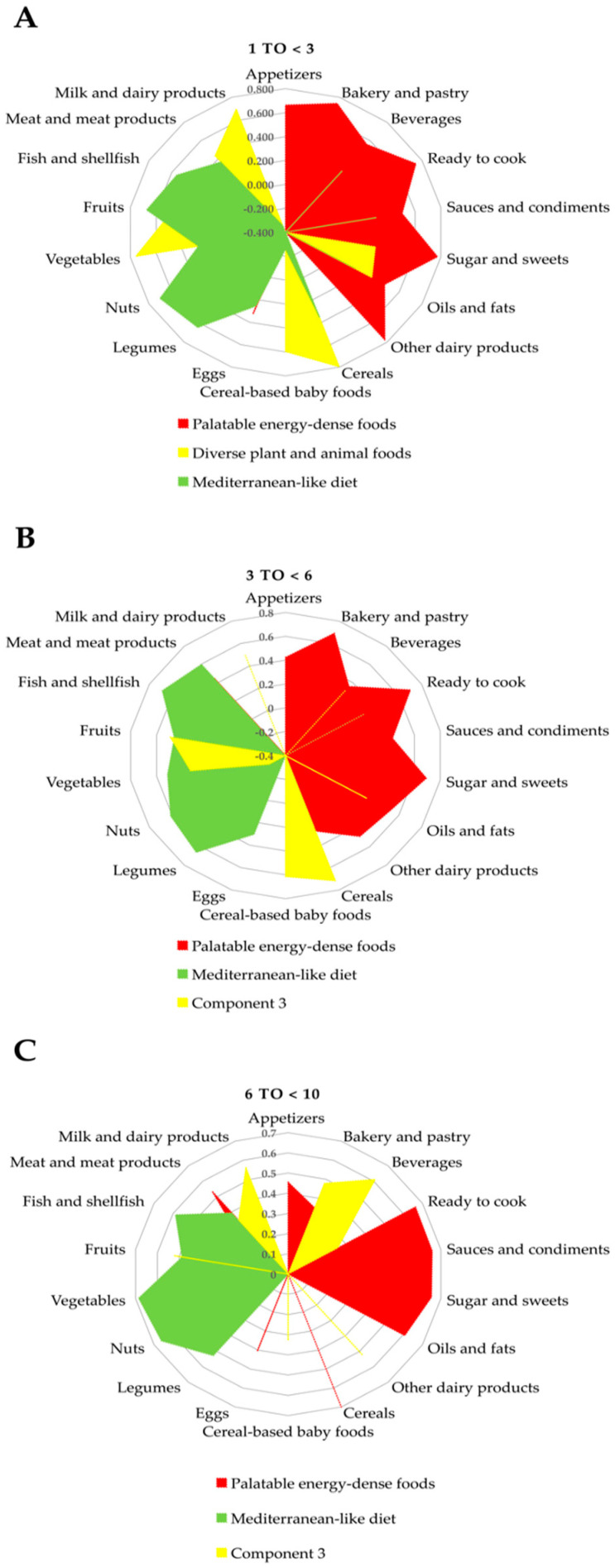
Dietary patterns extracted from principal component analysis of 18 major food groups in a representative cohort (SRS) of the EsNuPI (*n* = 740). (**A**). Children from 1 to <3 years (*n* = 169), (**B**). children from 3 to <6 years (*n* = 256), and (**C**). children from 6 to <10 years (*n* = 315).

**Figure 2:**
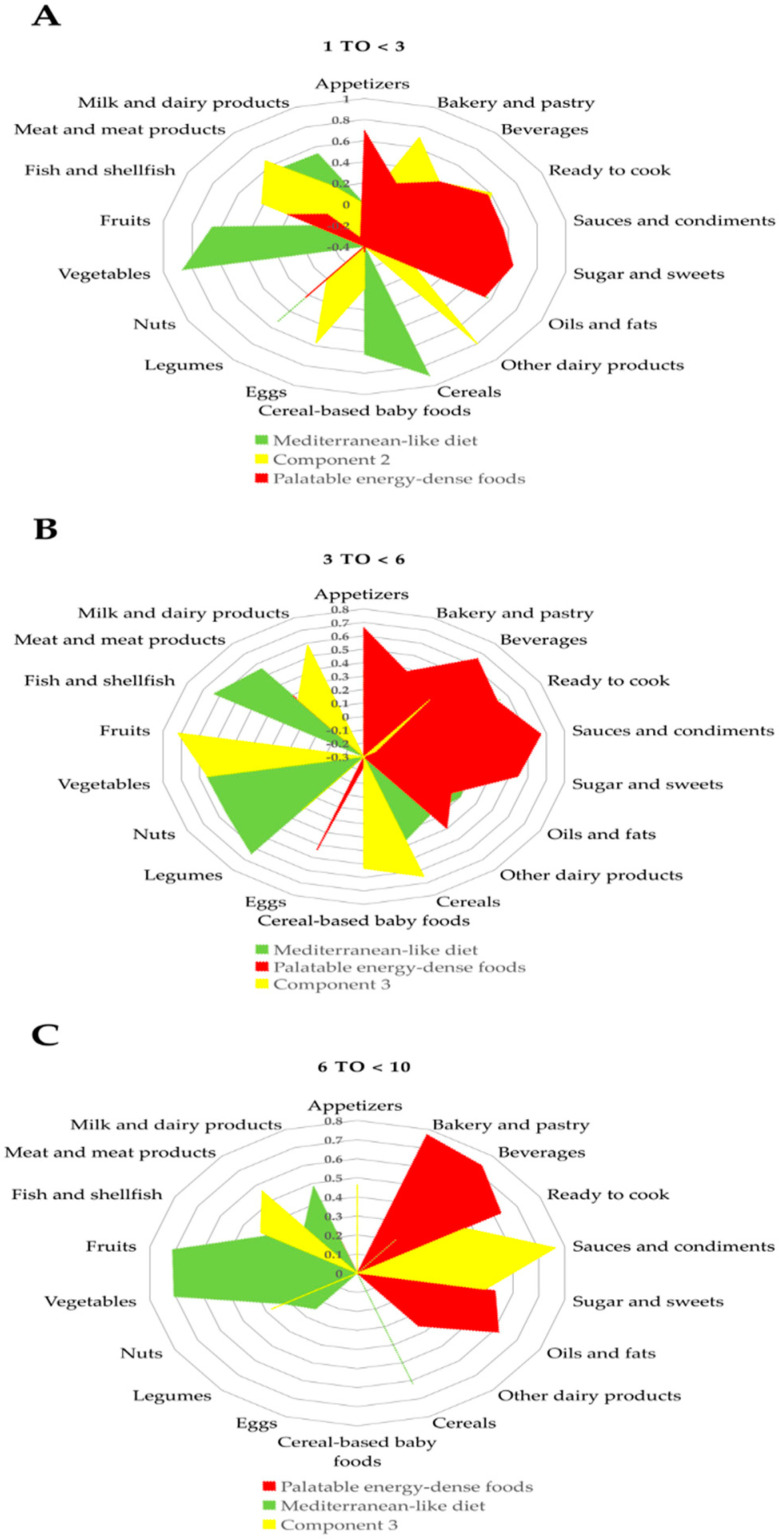
Dietary patterns extracted from the principal component analysis of 18 major food groups in a sample of adapted milk consumers cohort (AMS) for the EsNuPI (*n* = 772): (**A**). Children from 1 to <3 years (*n* = 302), (**B**). children from 3 to <6 years (*n* = 277), and (**C**). children from 6 to <10 years (*n* = 193).

DPs were computed using the PCA statistical approach. Bartlett’s tests of sphericity and KMO supported the appropriateness of factor analysis in both the SRS and AMS populations for the three different age groups. All Bartlett’s tests of sphericity were significant, and the KMO value was >0.626 in all analyses. Three major factors were extracted through PCA using the 18 food groups, as mentioned above, which explained from 37.56% to 51.26% of the variance in the six studied models (Table S1). Figure 1 and Figure 2 show those components as DPs for each sample and by age groups.

For the SRS, in children from 1 to <3 years, we observed three major factors or components: The first component (palatable energy-dense foods) had high positive loadings on sweetened dairy products, sugars and sweets, bakery and pastry, ready to cook, appetizers, beverages, sauces and condiments, and oils and fats, and negative loading on cereal-based baby foods. The second component (diverse plant and animal foods) was characterized by high positive loadings on cereals, vegetables, milk and dairy products, cereal-based baby foods, fruit, meat and meat products, and oils and fats. This pattern is close to the traditional Mediterranean diet pattern but with relatively high consumption of dairy and meat products. The third component (Mediterranean-like diet) had high positive loadings on nuts, fruits, legumes, cereals, fish and shellfish, and meat and meat products, and negative loading on milk and dairy products. Regarding children aged 3 to <6 years, we observed three major components: The first component was similar to children 1 to <3 years and was therefore defined as the palatable energy-dense foods component. The second component was described as the Mediterranean-like diet, and the third component had high positive loadings on cereals, cereal-based baby foods, fruit, vegetables, appetizers, and oils and fats. Likewise, in children aged 6 to <10 years there were three major components; again, the first component was characterized as palatable energy-dense foods. The second component was defined as Mediterranean-like diet and the third component was defined by beverages, milk and dairy products, fruits, other dairy products, bakery and pastry, meat and meat products, and cereal-based baby foods with high positive loadings (Figure 1 and Table S1).

Concerning the AMS, in children aged 1 to <3 years, we observed three major factors. The first component was characterized as Mediterranean-like diet. The second component was defined by high positive loading on other dairy products, bakery and pastry, meat and meat products, ready to cook, eggs, fish and shellfish, beverages and sauces and condiments. The third component was characterized as palatable energy-dense foods. Children aged 3 to <6 years had three major components: the first component was defined by Mediterranean-like diet, the second component was defined as palatable energy-dense foods, and the third component was composed of fruits, cereals, vegetables, milk and dairy products and cereal-based baby foods with high positive loadings. Finally, children aged 6 to <10 years had three major components: the first component was defined by palatable energy-dense foods, the second component was characterized as a Mediterranean-like diet, and the third component was defined by high positive loadings on sauces and condiments, meat and meat products, appetizers, ready to cook, sugars and sweets, fish and shellfish, and nuts (Figure 2 and Table S1).

In SRS, moderate–vigorous physical activity increased with age, whereas PAL and BMI z-score did not differ across the age groups. In AMS, children had a similar result in age and moderate–vigorous physical variables to SRS. However, PAL and BMI z-score were significantly different in the different age groups: PAL was significantly higher in children aged 6 to <10 years, and BMI z-score was significantly higher in children aged 1 to <3 years. Parental education and HI variables did not show any statistical differences in both samples across ages (Table 3). According to the overall comparison between SRS and AMS, SRS had higher values in age and moderate–vigorous physical activity, whereas PAL and BMI z-score did not show statistical differences. Across different ages, children aged 3 to <6 years in AMS had lower BMI z-scores than SRS children of the same age. Finally, the distribution in HI in children from AMS aged 1 to <3 years was significantly different from children in SRS of the same age (Table 3).

### 3.3. Dietary and Lifestyle Patterns

**Figure 3:**
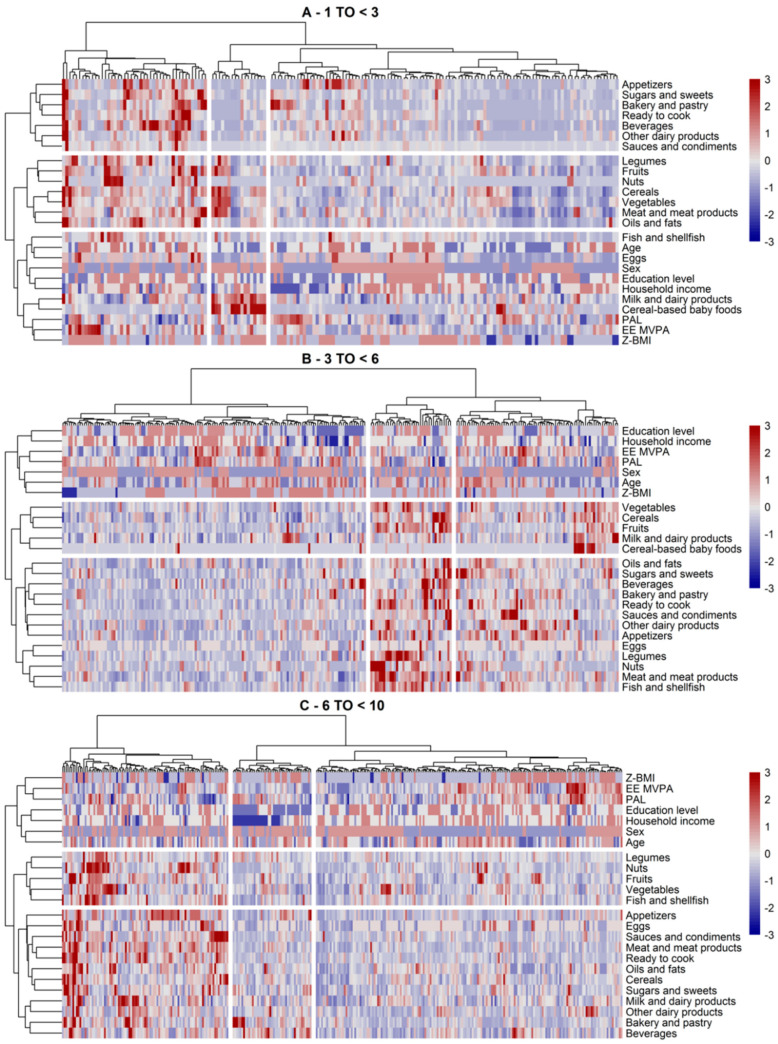
Clusters of subjects and dietary and lifestyle variables identified via hierarchical clustering in SRS by age groups ((**A**) 1–3 years, (**B**) 3–6 years, (**C**) 6 to <10 years) in the Nutritional Study in Spanish Pediatric Population (EsNuPI) (*n* = 740). The clusters are visually separated by longitudinal marks on vertical and horizontal faces (clusters of subjects or dietary/lifestyle variables, respectively). The vertical and horizontal dendrograms denote the relationship between the clusters, i.e., similar observations. The color bar refers to levels above (red) or below (blue) the mean intake of the dietary variable or mean scores of lifestyle variables. Increased color intensities indicate larger differences around the mean. Abbreviations: EE MVPA, energy expenditure moderate and vigorous physical activity; PAL, physical activity level; Z-BMI-for-age, body mass index for age z-score.

**Figure 4:**
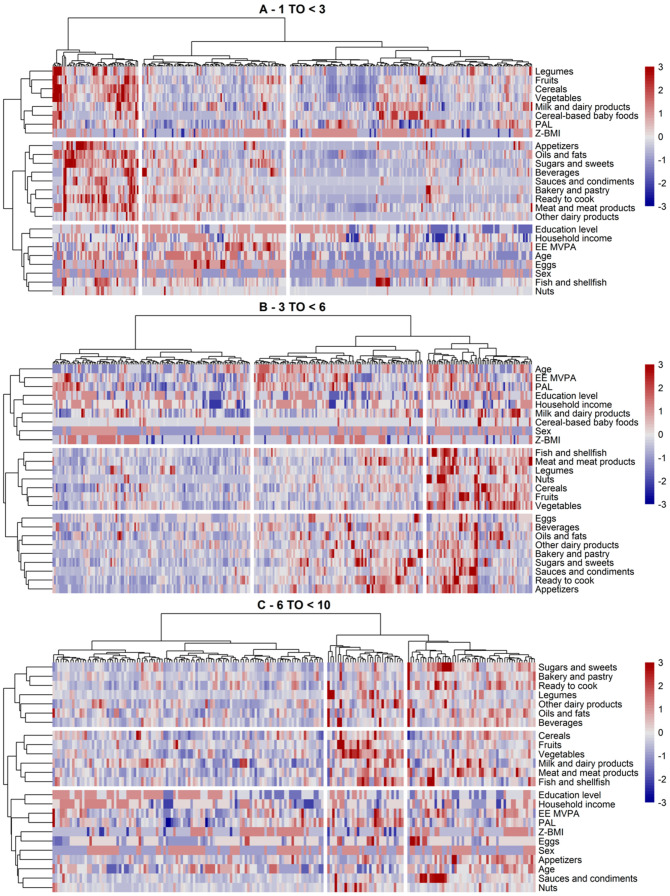
Clusters of subjects and dietary/lifestyle variables identified via hierarchical clustering in the AMS by age group ((**A**) 1–3 years, (**B**) 3–6 years, (**C**) 6 to <10 years) in the Nutritional Study in Spanish Pediatric Population (EsNuPI) (*n* = 772). The clusters are visually separated by longitudinal marks on vertical and horizontal faces (clusters of subjects or dietary/lifestyle variables, respectively). The vertical and horizontal dendrograms denote the relationship between the clusters, i.e., similar observations. The color bar refers to levels above (red) or below (blue) the mean intake of dietary variable or mean scores of lifestyle variables. Increased color intensities indicate larger differences around the mean. Abbreviations: EE MVPA, energy expenditure moderate and vigorous physical activity; PAL, physical activity level; Z-BMI-for-age, body mass index for age z-score.

Clusters of subjects within each study sample, classified based on their dietary and lifestyle characteristics, are shown in Figure 3 for SRS and in Figure 4 for AMS. Overall, there were three main clusters of subjects and dietary/lifestyle variables identified by the clustering algorithm (the silhouette method suggested three to four clusters by rows and columns). This was the case in both study samples and for every age group.

In the youngest age group in the SRS cohort (Figure 3A), we found three clusters of subjects and dietary/lifestyle variables. Regarding the dietary/lifestyle clusters (in rows), cluster 1 contained more energy-dense foods; cluster 2 grouped foods of vegetable origin (legumes, fruits, nuts, cereals, and vegetables), meat and meat products, as well as oils and fats (similar to the “diverse plant and animal foods” and Mediterranean-like PCAs); and cluster 3 featured intake of milk and dairy products, eggs, fish, and cereal-based baby foods. In terms of the subject’s characteristics, the first cluster was defined by older children with higher intakes of all food groups, except cereal-based baby foods. These subjects seemed to have a higher BMI z-score and high moderate–vigorous physical activity. Conversely, subjects in the second cluster showed a higher intake of food groups of dietary cluster 2, as well as milk and dairy products and cereal-based baby foods. These subjects were more likely to be younger (around one year old) and tended to have a higher household income than the average. In the third cluster, we observed somewhat mixed patterns with regard to dietary and lifestyle variables. Subjects of this cluster were more inclined to consume fewer amounts of the food groups in dietary cluster 2, whereas some subjects showed both high and low intakes of the food groups in dietary cluster 1 (the more palatable energy-dense food cluster). Within this cluster of subjects, those with lower intakes of all food groups (below their mean intake) seemed to have a lower BMI z-score, higher physical activity, and a higher household income (and parental educational level). The opposite was observed for subjects whose intake of energy-dense foods (mostly appetizers and bakery and pastry) tended to be high. There were no clear patterns for any other lifestyle variables.

Within AMS (Figure 4A), we observed similar dietary clusters, except that cereal-based baby foods and milk and dairy products were clustered with legumes, cereals, fruits, and vegetables. Other dairy products were still included in the more palatable energy-dense dietary cluster. Clusters of subjects were also similar, with one cluster group of older children showing a rather high consumption of all foods including cereal-based baby foods, a second cluster group of older-aged but less active (overall PAL) children whose consumption of energy-dense foods exceeded that of the healthier dietary cluster, and a third cluster mostly composed of toddlers and with a distinguishable subcluster of milk, dairy, and cereals consumers. This latter subcluster seemed to comprise children with a higher BMI z-score. Parental income and education also appeared to be correlated with these subclusters.

In the three to six years age group, we observed similar dietary clusters in both study samples (Figure 3B and Figure 4B). The main difference between the two was that legumes, nuts, and fish were included in the energy-dense food group in SRS, whereas these food groups clustered together with other plant-based foods in AMS. Milk and dairy products and cereal-based baby foods tended to cluster within this healthier cluster too. Children of this age group showed either high, low, or mixed consumption patterns of all foods. In the mixed group, we observed two distinct groups of children eating either higher amounts of healthy foods (i.e., plant-based) or less healthy foods (i.e., more palatable energy-dense foods). These subgroups were shaped by the children’s age and parental income and education. For instance, those with higher mean intake of plant-based foods and low consumption of energy-dense foods were younger and tended to have higher family incomes.

The children aged six to nine years were similar to the above-described clusters and in the two study samples (Figure 3C and Figure 4C), with milk, dairy products, and cereals being clustered with the palatable energy-dense foods in SMS or fruits and vegetables in AMS. The clusters of children (the high-, low-, and mixed-level consumers) also held in these study samples. The mixed group of both study samples mostly included children of lower parental income and education, showing a somewhat higher consumption of the more energy-dense foods.

Differences between the clusters with regard to dietary and lifestyle variables, to complement the description of the above clusters, are shown in Table 4. The results showed significant differences by age in all clusters and both study cohorts, except for SRS aged three to six years (data not shown). Although potential subclusters were not considered as a sample descriptive, we observed several important differences between the clusters. We found significant differences between the cluster of subjects by levels of physical activity. In general, lower activity levels in moderate–vigorous activity (EE-MVPA) and overall physical activity (PA) variables were related to unhealthier dietary choices, e.g., the second cluster in SRS aged 6 to <10 years, comprising subjects with higher consumption of palatable energy-dense foods, showed lower levels of vigorous activity compared to the other clusters. The same was observed in AMS for the third cluster and PAL. BMI z-scores were significantly lower in some clusters compared to the others, e.g., in the third cluster in children aged less than three years in SRS and in children aged over six years in AMS. Parental education (and HI) also differed significantly across the clusters in some age groups. For example, younger-aged children in AMS whose parents were of lower educational level were more frequently in the third cluster which mainly featured a lower consumption of milk and dairy products and cereal-based baby foods. Similarly, children aged 6 to <10 years in SRS with low parenteral education levels were more common in the second cluster, which indicated a higher consumption of sweetened beverages, pastries, and other energy-dense foods.

## 4. Discussion

The main goals of this study were to determine the DPs in a representative SRS in three different age groups of Spanish children (one to nine years old) and in a convenience cohort of AMS of the same age groups, and to examine how several sociodemographic and lifestyle factors are clustered within the DPs and among the three age groups. For SRS, we observed three components in the different age groups. The first component was “palatable energy-dense foods” in all group ages; here, the consumption of sweetened dairy products, sugars and sweets, bakery and pastry, ready to cook, appetizers, beverages, sauces and condiments, and oils and fats products predominated. The second and third components were a mix of Mediterranean-like diet and diverse plant and animal foods. Regarding AMS children, component one was a Mediterranean-like diet in children aged 1 to <3 years old, and 3 to <6 years old, whereas children aged 6 to <10 years were similar to SRS with palatable energy-dense foods. Similar to SRS, components two and three were a mix of palatable energy-dense foods and the Mediterranean-like diet, respectively.

Regarding the dietary and lifestyle clusters, we observed dietary clusters that were, to some extent, similar to the DPs observed in the PCA. In brief, in the youngest SRS children (aged 1 to <3 years), we observed a clustering of the more energy-dense foods (cluster 1) that resembled the palatable processed food cluster derived with PCA, a more Mediterranean-like group of foods (cluster 2), and a dairy milk products cluster including cereal-based baby foods (cluster 3), both of which tended to represent the Mediterranean-diet-like PCAs. The children´s age and their lifestyle characteristics were found to be correlated with these dietary clusters. While the Mediterranean diet clusters were always present, some variations were observed in the older age groups, likely due to dietary adaptations to children´s growth. As such, there were not only slightly different dietary clusters defined in these other age groups, but also groups of children who were high- or low-level consumers of all foods. Within AMS, we observed somewhat similar dietary clusters to those seen in SRS, although some foods clustered differently, and the Mediterranean diet-like dietary pattern tended to be ranked first. It is important to mention that age was one of the main variables in our study and the differences were analyzed within the groups of the same ages to avoid comparisons determined with the stage of life (e.g., children with 1 to <3 years and 6 to <10 years). We also took special consideration of the fact that food groups, such as cereal-based baby foods, were more likely consumed by children aged less than 3 years.

The importance of adequate nutrition in early childhood development has been recognized for many decades. Epidemiological analyses and animal studies showed that nutritional effects early in life can encourage the responsiveness of the body to have a better nutritional status in the adult life [46]. Infants develop rapidly from 6 to 24 months and have high nutrient needs in comparison to their body size, so they might be disposed to dietary disproportions and insufficiencies [47]. However, the dietary intake, nutritional status, and lifestyle patterns of infants and toddlers are less well-characterized compared to children aged 9 to 12 years, adolescents, and adults [1,37,48,49,50].

Knowing dietary habits and their determinants is essential for evaluating the adequacy of nutrient intake as well as its impact on health in children. Overweight and obesity among children are a major health issue, and the diets of many children exceed dietary guidelines for fat, cholesterol, added sugar, saturated fatty acids, and sodium, and are low in fiber [51]. Changes from undernutrition to overnutrition are occurring in most nations in the world, with the trend of children consuming higher intakes of nutrients such as sugar, salt, and saturated fat [52]. Therefore, several studies attempted to characterize dietary patterns and associated factors. Dietary intake patterns in 3427 children aged six years from Brazil were strongly influenced by socioeconomic characteristics. Younger maternal age at birth and both early weaning and early introduction of complementary feeding appeared to be related to unhealthier patterns with snacks and treats, including consumption of candies, sweetened beverages, chips/crisps, bread, butter, mayonnaise, margarine, coffee, and sugar [33]. Another study applied PCA to derive DPs in two Australian studies of 14-month-old (*n* = 552) and 24-month-old (*n* = 493) children. Two patterns were identified in these two studies: one was characterized by fruits, grains, vegetables, cheese, and nuts, and the second by white bread, spreads, sweetened beverages, snacks, chocolate, and processed meat. Lower maternal age and early breastfeeding termination were connected with the second component, and patterns were not related to BMI z-score [53]. A systematic review showed that in the majority of Mediterranean countries, children aged one to five years consumed fruit and vegetables quite frequently, but also notably consumed sugared beverages and snacks [54]. A recent study in 525 children aged two to five years from Lebanon reported two DPs, with the first described as “fast food and sweets” with higher consumption of sweetened beverages, fast foods, salty snacks and sweets, and a second pattern denominated as “traditional Lebanese”, with higher intakes of cereals, dairy products, fruits, and vegetables [55]. These previous studies highlighted the remarkably unhealthy DPs in children, similar to our results. In SRS, we identified palatable energy-dense foods as the main component, with sweetened dairy products, sugars and sweets, bakery and pastry, ready to cook, appetizers, beverages, sauces and condiments, and oils and fats products prevailing in the three age groups.

The term “Mediterranean diet” has been extensively used to explain the traditional dietary habits of people living around the Mediterranean Sea. This diet is defined by the predominant consumption of fruits, vegetables, whole grain cereals, legumes, nuts and seeds, and fish, with olive oil as the main source of added fat. Other important features of the Mediterranean diet are a relatively low intake of dairy products, mainly as fermented milk, i.e., yogurt and cheese, and low to moderate intake of fish and poultry meat, low consumption of red meat, and modest wine drinking in adults [56,57]. Evidence from epidemiological studies supports the cardiometabolic benefits of the Mediterranean diet in terms of its favorable effects on obesity, high blood pressure, type 2 diabetes mellitus, and dyslipidemia [58]. The Mediterranean diet has been recognized as one of the most important DPs related to human health [58]. A recent umbrella review including 29 meta-analyses with 12,800,000 individuals showed that adherence to this dietary pattern is correlated with a significant risk reduction in all-cause mortality, cardiovascular and coronary heart disease, myocardial infarction, cancer, neurodegenerative diseases, and type 2 diabetes mellitus incidence [59,60]. In particular, the Mediterranean diet is the dietary pattern most recommended to reduce cardiovascular risk in different populations by some scientific societies, such as the European Society of Cardiology, the European Atherosclerosis Society, the Canadian cardiovascular society, and the American Heart Association Nutrition Committee, among others [60]. Specifically, in children, adherence to the Mediterranean diet is associated with a lower likelihood of presenting excess body weight and obesity [61,62]. In our study, the most consistent dietary pattern in children in SRS was related to a high consumption of energy-dense foods. However, secondary patterns resembled those of a Mediterranean-like diet. In contrast, AMS children exhibited a dietary pattern related to the Mediterranean diet as the main component in the majority of the studied population, with higher consumption of cereals, fruits, and vegetables, and milk and dairy products.

A total of 9301 European children aged two to nine years participated at baseline and two-year follow-up examinations of the “Identification and prevention of dietary- and lifestyle-induced health effects in children and infants” (IDEFICS) study. For the Spanish population, three DPs were recognized at baseline and follow-up by applying clustering methods. These DPs were based on a higher frequency of consumption of snacks and fast food (processed), sweet foods and drinks (sweet), and fruits and vegetables (healthy) [35]. In Spain, the ANIBES study also demonstrated the appearance of three DPs in 207 children from 9 to 12 years, and 208 adolescents from 13 to 17 years with a low physical activity and poorer diet lifestyle pattern, and a high physical activity, low sedentary behavior, longer sleep duration, healthier diet lifestyle pattern [37]. In our study, those three components were palatable energy-dense foods and a Mediterranean-like diet. Changes in PAL and BMI z-score were observed, but only in the AMS cohort. To the best of our knowledge, those are the only studies reporting DPs based on Spanish data.

Several studies showed that the ingestion of dairy foods has a neutral or inverse association with adverse cardiometabolic health outcomes [12,13]. Cereal consumption might be linked to a beneficial long-term health effect by providing non-digestible carbohydrates, which are mainly responsible for the development of an adult-like microbiota by increasing the *Bacteroides* population [63]. A study reported a negative association between cereal intake and hepatic fat in 110 overweight/obese children, stating that cereal ingestion might decrease hepatic fat in this pediatric population [64]. This healthier dietary pattern appeared mainly in the AMS cohort and could be related to the consumption of milk or adapted milk with cereals in the early stages of life. Interestingly, this dietary pattern was also related to the consumption of healthier foods, including cereals, vegetables and fruits.

The results of our cluster analysis combining lifestyle and dietary factors were consistent with those reported by other studies. These studies reported a healthy energy-balance-related behavior pattern characterized by a healthier diet with high levels of physical activity and low levels of sedentary behavior between children in different countries. Other studies conducted in different countries also reported the clustering of a combination of sedentary lifestyle with healthy diet [28,29,30,31,32,65]. In Spanish studies conducted within the ANIBES study (455 children and adolescents aged older than nine years), DPs, physical activity, and sleeping patterns were shown to be clustered in two different groups: a low physical activity and poorer diet lifestyle pattern, which included a higher proportion of girls, and a healthier diet lifestyle pattern that was defined by high physical activity with low sedentary behavior and longer sleep duration [37]. We considered a younger population, so this study is therefore the first report of dietary/lifestyle clusters in Spanish toddlers and teenagers. Although our study population had a number of specific features, we found that poorer lifestyle and unhealthy dietary patterns were grouped together (for example, the more energy-dense foods cluster with lower physical activity levels). In contrast, the healthier dietary patterns were more likely present among children with healthier lifestyle patterns (for example, the more Mediterranean-like cluster with higher physical activity levels). Within age groups, we observed that age had a strong impact on the clustering, with dietary clusters and levels of food consumption being age-determinant. Importantly, lower household income and parental education seemed to be correlated with unhealthy dietary clusters. Children from low socioeconomic status seemed to be more likely to present sedentary behaviors and low physical activity levels, though not all studies agree in this regard [66]. As mentioned above, in our study, we observed that the unhealthier dietary clusters were more frequently maintained by children with lower physical activity levels. There were apparent differences between SRS and AMS; the latter was a convenience sample of mostly formula-fed children. However, these differences were, for the most part, not significant. We observed concordant clusters between both study samples, which partly validates our findings.

The major strength of this study was the opportunity to investigate a large sample of Spanish children representative of Spanish children aged 1 to <10 years living in urban areas, and the possibility of comparing the results in a convenience sample of children (AMS) of the same age group. Two different methodological approaches were applied to identify the DPs and to validate our findings: PCA and cluster analyses (bivariate and hierarchical clustering). We relied upon dietary information collected via the FFQ and 24 h dietary recalls, but we considered the FFQ dietary data in the present study because a higher variance was explained and uses almost the total EsNuPI sample (1512/1514).

The limitations of the present study were the cross-sectional design, which provides evidence for the relationship between lifestyle and dietary factors but does not allow for causal inference. Residual confounding by unobserved and unmeasured factors is probable, and information on dietary intake, physical activity, weight, and other variables is prone to measurement error since the data were self-reported by the participants. However, a careful multistep quality control procedure was implemented to derive dietary intake to minimize this bias. For instance, the interviewers were coached by knowledgeable dieticians/nutritionists, and these professionals were responsible for inspection of the food consumption records during the study process and from the coding process to verify the survey data.

Subjective decisions have been constantly reported as a limitation in studies deriving a posteriori DPs with PCA or cluster analysis [23]. However, based on the available evidence, most identified DPs showed good reproducibility, fair relative validity, and good construct validity across different statistical solutions [24].

It is unlikely that energy intake influenced our results according to earlier studies comparing DPs derived with and without energy adjustment [67]. Another limitation is related to non-rural information due to the study design; we only analyzed areas with more than 50,000 inhabitants. The size of the study population for reliably assessing DPs was probably limited in some ages groups. Finally, the use of FFQ in this kind of population has some limitations and requires parental supervision. Children were with the parents when the study questionnaires were answered; whenever it was possible, we preferred that children answered the questions themselves and their parents only assisted with some answers to avoid loss of information.

## 5. Conclusions

The current study is the first exploring dietary clusters in Spanish children aged under nine years to the highest extent possible. The three identified dietary clusters demonstrated the existence of an unhealthy dietary pattern, with palatable and processed foods (also defined as palatable energy-dense foods) being consumed in all age groups and in the two study samples. Two Mediterranean-like DPs (the more healthier DPs) were also identified. Children from AMS showed a dietary pattern related to the Mediterranean diet as the main component, with high consumption of cereals, fruits, and vegetables, as well as milk and dairy products. Certain lifestyle and sociodemographic factors, i.e., PAL, parental education, and HI, were found to be significantly correlated with the dietary clusters, suggesting that physical activity and socioeconomic status may be to some extent determinants of these DPs. Thus, the findings provide guidance for designing lifestyle interventions based on age group or based on parent education that could reverse the appearance of unhealthy DPs in the child population. Our findings represent a wake-up call for regulatory bodies and professional organizations to try to reduce the consumption of energy-dense foods. Further research is warranted to evaluate how these variables or others influence the health status of children and to assess the whole picture of the relationships between consumption of nutrients and lifestyle factors in properly designed longitudinal studies including the whole population (urban and rural). Intervention studies considering dietary patterns such as the Mediterranean diet and promotion of physical activity might be necessary to establish the role of dietary and lifestyle factors on health determinants of the Spanish pediatric population.

## Figures and Tables

**Table 1:** Distribution of the studied sample in the Nutritional Study in Spanish Pediatric Population (EsNuPI) (*n* = 1512) *.

		Whole Population (*n* = 1512)	Spanish Reference Cohort (SRS) (*n* = 740)	Adapted Milk Consumers Cohort (AMS) (*n* = 772)
Sex	Boys	757	372	385
	Girls	755	368	387
Age (years)	1 to <3	471	169	302
	3 to <6	533	256	277
	6 to <10	508	315	193

* Distribution of the studied sample in the EsNuPI study within subjects with complete food frequency questionnaire (FFQ) data as well as information on the variables of interest.

**Table 2:** Food group consumption according to food frequency questionnaires in the EsNuPI study (*n* = 1512).

Food Groups	Spanish Reference Cohort (SRS)				Adapted Milk Consumers Cohort (AMS)			
	Total	1 to <3 Years	3 to <6 Years	6 to <10 Years	Total	1 to <3 Years	3 to <6 Years	6 to <10 Years
	(*n* = 740)	(*n* = 169)	(*n* = 256)	(*n* = 315)	(*n* = 772)	(*n* = 302)	(*n* = 277)	(*n* = 193)
Milk and dairy products (g/day)	608.9 ± 308.2	738.8 ± 368.6 ^a^	588.9 ± 275.2 ^b^	555.5 ± 277.5 ^b^	682.6 ± 320.6 *	820.5 ± 342.6 ^a,^*	604.1 ± 267.7 ^b^	579.6 ± 276.4 ^b^
Cereals (g/day)	138.5 ± 77.6	145.2 ± 84.6	134.1 ± 63.7	138.5 ± 83.7	143.3 ± 80.4	158.0 ± 102.5 ^a^	133.9 ± 64.2 ^b^	133.9 ± 54.8a ^b^
Meat and meat products (g/day)	94.5 ± 58.1	74.3 ± 45.9 ^a^	93.5 ± 51.6 ^b^	106.2 ± 65.5 ^b^	88.6 ± 57.0 *	74.9 ± 59.1 ^a^	93.3 ± 52.4 ^b^	103.1 ± 55.5 ^b^
Oils and fats (g/day)	24.2 ± 19.8	21.7 ± 19.8 ^a^	24.2 ± 18.9 ^b^	25.7 ± 20.4 ^b^	23.6 ± 16.6	20.8 ± 15.5 ^a^	23.2 ± 15.0 ^a^	28.6 ± 19.2 ^b,^*
Bakery and pastry (g/day)	21.4 ± 26.6	12.4 ± 20.1 ^a^	20.8 ± 26.2 ^b^	26.6 ± 28.6 ^c^	17.4 ± 26.0 *	9.7 ± 18.7 ^a^	18.9 ± 24.3 ^b^	27.4 ± 33.5 ^c^
Fruits (g/day)	253.1 ± 196.1	293.6 ± 216.6 ^a^	256.5 ± 209.8 ^ab^	228.5 ± 168.0 ^b^	264.6 ± 207.9	302.4 ± 226.6 ^a^	243.0 ± 200.1 ^b^	236.4 ± 178.3 ^b^
Vegetables (g/day)	130.9 ± 106.6	177.0 ± 146.5 ^a^	128.0 ± 97.4 ^b^	108.6 ± 76.8 ^c^	149.7 ± 120.7 *	190.5 ± 153.1 ^a^	128.4 ± 87.7 ^b^	116.3 ± 79.1 ^b^
Other dairy products (g/day)	73.5 ± 79.7	51.3 ± 85.4 ^a^	73.2 ± 66.6 ^b^	85.7 ± 84.0 ^b^	61.3 ± 77.0 *	38.5 ± 79.7 ^a^	71.1 ± 74.9 ^b^	83.2 ± 65.7 ^c^
Sugars and sweets (g/day)	40.9 ± 39.5	30.5 ± 40.8 ^a^	44.4 ± 42.2 ^b^	43.6 ± 35.5 ^b^	34.2 ± 31.8 *	23.6 ± 25.8 ^a,^*	37.4 ± 30.6 ^b^	46.3 ± 36.7 ^c^
Ready to cook (g/day)	23.3 ± 23.8	11.8 ± 17.4 ^a^	22.6 ± 20.8 ^b^	30.1 ± 26.5 ^c^	19.4 ± 18.9 *	9.6 ± 12.8 ^a^	21.5 ± 16.8 ^b^	31.5 ± 21.7 ^c^
Eggs (g/day)	23.3 ± 20.5	17.9 ± 18.0 ^a^	24.4 ± 24.3 ^b^	25.2 ± 17.7 ^b^	20.7 ± 15.8 *	16.1 ± 14.0 ^a^	22.6 ± 16.9 ^b^	25.1 ± 15.0 ^b^
Legumes (g/day)	21.0 ± 17.6	21.1 ± 19.8	20.9 ± 14.5	21.0 ± 18.6	20.6 ± 17.6	19.6 ± 18.2 ^a^	20.1 ± 12.7 ^ab^	22.9 ± 22.1 ^b^
Fish and shellfish (g/day)	40.1 ± 37.8	28.6 ± 38.9 ^a^	38.9 ± 34.5 ^b^	47.2 ± 38.2 ^c^	33.7 ± 28.6 *	25.8 ± 26.0 ^a^	36.5 ± 30.6 ^b^	41.9 ± 26.8 ^c^
Beverages (g/day)	171.9 ± 163.9	112.8 ± 138.3 ^a^	189.2 ± 178.6 ^b^	189.5 ± 157.1 ^b^	163.3 ± 181.6 *	126.8 ± 163.6 ^a^	156.6 ± 144.3 ^b,^*	230.0 ± 232.0 ^c^
Cereal-based baby foods (g/day)	1.8 ± 6.3	5.9 ± 10.7 ^a^	1.1 ± 4.7 ^b^	0.08 ± 0.8 ^c^	3.4 ± 9.4 *	7.6 ± 12.6 ^a,^*	1.2 ± 6.4 ^b^	0.02 ± 0.2 ^c^
Appetizers (g/day)	14.8 ± 16.0	9.3 ± 13.4 ^a^	16.0 ± 16.5 ^b^	16.8 ± 16.3 ^b^	14.0 ± 16.3 *	9.1 ± 14.1 ^a^	15.0 ± 14.4 ^b^	20.5 ± 19.2 ^c,^*
Sauces and condiments (g/day)	4.3 ± 7.6	2.4 ± 9.5 ^a^	4.7 ± 7.7 ^b^	5.0 ± 6.2 ^c^	3.7 ± 6.5 *	1.6 ± 3.9 ^a^	4.2 ± 6.6 ^b^	6.4 ± 8.4 ^c,^*
Nuts (g/day)	3.7 ± 5.8	1.7 ± 4.5 ^a^	3.3 ± 4.9 ^b^	5.1 ± 6.7 ^c^	3.1 ± 7.7 *	1.2 ± 5.9 ^a^	3.2 ± 6.1 ^b^	5.7 ± 10.8 ^c^

Data are expressed as mean ± standard deviation. Differences among age groups within the same cohorts (SRS or AMS) were calculated using an H Kruskal–Wallis test corrected by Bonferroni post hoc test. Significant differences among age groups within samples are expressed with different superscript letters (*p* < 0.05). * Differences between SRS and AMS for the considered age groups were calculated using the Mann–Whitney U test.

**Table 3:** Sociodemographic variables in the Nutritional Study in Spanish Pediatric Population (EsNuPI; *n* = 1512).

Variables	Spanish Reference Cohort (SRS)				Adapted Milk Consumers Cohort(AMS)			
	Total	1 to <3 Years	3 to <6 Years	6 to <10 Years (*n* = 315)	Total	1 to <3 Years	3 to <6 Years	6 to <10 Years
	(*n* = 740)	(*n* = 169)	(*n* = 256)		(*n* = 772)	(*n* = 302)	(*n* = 277)	(*n* = 193)
**Energy expenditure Moderate–vigorous physical activity (minutes/day)**	402.9 ± 282.0	236.4 ± 226.0 ^a^	392.1 ± 238.5 ^b^	501.6 ± 298.1 ^c^	356.8 ± 273.1 *	235.2 ± 199.1 ^a^	357.0 ± 196.3 ^b^	546.4 ± 350.1 ^c^
PAL (per 24 h)	1.6 ± 0.6	1.6 ± 0.3	1.6 ± 0.2	1.6 ± 0.2	1.6 ± 0.3	1.5 ± 0.3 ^a^	1.5 ± 0.2 ^a^	1.6 ± 0.2 ^b^
BMI-for-age z-score	0.6 ± 1.8	0.7 ± 1.7	0.7 ± 2.1	0.4 ± 1.7	0.6 ± 1.8	1.0 ± 2.1 ^a^	0.4 ± 1.7 ^b,^*	0.4 ± 1.4 ^b^
**Parental education**								
a. Low (less than 10 years of education; the primary school or less)	22.0% (163)	26.6% (45)	18.4% (47)	22.5% (71)	18.5% (143)	18.5% (56)	17.0% (47)	20.7% (40)
b. Medium (12 years of education; higher secondary education)	44.2% (327)	36.7% (62)	45.7% (117)	47.0% (148)	42.2% (326)	39.4% (119)	44.4% (123)	43.5% (84)
c. High (13 years or more of education; higher vocational, college and university studies)	33.8% (250)	36.7% (62)	35.9% (92)	30.5% (96)	39.3% (303)	42.1% (127)	38.6% (107)	35.8% (69)
**Household income**								
a. Low	9.6% (71)	13.6% (23)	6.3% (16)	10.2% (32)	9.0% (69)	8.6% (26) *^.^*	9.0% (25)	9.3% (18)
b. Mid-low	14.3% (106)	15.4% (26)	16.4% (42)	12.1% (38)	12.7% (98)	8.9% (27) **	13.0% (36)	18.1% (35)
c. High (mid-mid, mid-high and high)	49.4% (366)	43.8% (74)	52.3% (134)	50.1% (158)	50.1% (387)	53.0% (160) **	48.4% (134)	48.2% (93)
d.- DK/NA/REF	26.7% (197)	27.2% (46)	25.0% (64)	27.6% (87)	28.2% (218)	29.5% (89) **	29.6% (82)	24.4% (47)

Data are expressed as mean ± standard deviation. Differences among age groups within the same cohorts (SRS or AMS) were calculated using an H Kruskal–Wallis test corrected by Bonferroni post hoc test. Significant differences among age groups within samples are expressed with different superscript letters (*p* < 0.05). * Differences between SRS and AMS for the considered age groups were calculated using the Mann–Whitney U test. ** A chi-squared test was used to compare the percentage differences between parental education and household income variables in SRS and AMS children, SRS vs. AMS and the age comparison. Abbreviations: BMI-for-age, body mass index for age; DK, do not know; NA, not available; PAL, physical activity level; REF, refusal.

**Table 4:** Descriptive characteristics of the clusters (CLU) of EsNuPI children by lifestyle and sociodemographic factors in SRS and AMS (dietary factors differed significantly across the clusters).

		**SRS 1 to <3 years**							**AMS 1 to <3 years**						
		**CLU3 (*n* = 108)**		**CLU1 (*n* = 45)**		**CLU2 (*n* = 17)**		***p*-Value**	**CLU3 (*n* = 155)**		**CLU 1 (*n* = 55)**		**CLU2 (*n* = 92)**		***p*-Value**
EE-MVPA		176 ± 152		370 ± 313		268 ± 197		<0.001	182 ± 145		228 ± 188		328 ± 247		<0.001
PAL		1.59 ± 0.31		1.51 ± 0.32		1.58 ± 0.29		0.341	1.57 ± 0.32		1.59 ± 0.27		1.44 ± 0.25		0.002
Education								0.283							<0.001
	Low	32	−29.60%	9	−20.00%	5	−29.40%		47	−30.30%	4	−7.27%	5	−5.43%	
	Medium	37	−34.30%	16	−35.60%	9	−52.90%		61	−39.40%	34	−61.80%	24	−26.10%	
	High	39	−36.10%	20	−44.40%	3	−17.60%		47	−30.30%	17	−30.90%	63	−68.50%	
Household Income								0.849	.						<0.001
	Low	17	−15.70%	5	−11.10%	1	−5.88%		21	−13.50%	3	−5.45%	2	−2.17%	
	Mid-low	14	−13.00%	8	−17.80%	4	−23.50%		12	−7.74%	5	−9.09%	10	−10.90%	
	High	46	−42.60%	20	−44.40%	8	−47.10%		82	−52.90%	36	−65.50%	42	−45.70%	
	DK/REF	31	−28.70%	12	−26.70%	4	−23.50%		40	−25.80%	11	−20.00%	38	−41.30%	
Z-BMI-for-age		2.28 ± 0.62		2.42 ± 0.54		2.65 ± 0.49		0.04	2.41 ± 0.60		2.33 ± 0.51		2.37 ± 0.61		0.675
		**SRS 3 to <6 years**							**AMS 3 to <6 years**						
		**CLU1 (*n* = 142)**		**CLU3 (*n* = 76)**		**CLU2 (*n* = 38)**		***p*-Value**	**CLU1 (*n* = 116)**		**CLU2 (*n* = 99)**		**CLU3 (*n* = 62)**		***p*-Value**
EE MVPA		372 ± 249		447 ± 233		356 ± 191		0.053	318 ± 186		387 ± 209		382 ± 185		0.017
PAL		1.6 ± 0.21		1.52 ± 0.22		1.49 ± 0.25		0.006	1.49 ± 0.22		1.56 ± 0.21		1.57 ± 0.23		0.014
Education								0.139							0.063
	Low	33	−23.20%	10	−13.20%	4	−10.50%		17	−14.70%	24	−24.20%	6	−9.68%	
	Medium	59	−41.50%	41	−53.90%	17	−44.70%		52	−44.80%	45	−45.50%	26	−41.90%	
	High	50	−35.20%	25	−32.90%	17	−44.70%		47	−40.50%	30	−30.30%	30	−48.40%	
Household Income								<0.001							0.168
	Low	11	−7.75%	4	−5.26%	1	−2.63%		12	−10.30%	6	−6.06%	7	−11.30%	
	Mid-low	19	−13.40%	18	−23.70%	5	−13.20%		11	−9.48%	13	−13.10%	12	−19.40%	
	High	63	−44.40%	43	−56.60%	28	−73.70%		62	−53.40%	43	−43.40%	29	−46.80%	
	DK/REF	49	−34.50%	11	−14.50%	4	−10.50%		31	−26.70%	37	−37.40%	14	−22.60%	
Z-BMI-for-age		2.36 ± 0.59		2.21 ± 0.50		2.29 ± 0.46		0.158	2.23 ± 0.57		2.24 ± 0.52		2.11 ± 0.48		0.266
		**SRS 6 to <10 years**							**AMS 6 to <10 years**						
		**CLU2 (*n* = 45)**		**CLU1 (*n* = 95)**		**CLU3 (*n* = 175)**		***p*-Value**	**CLU1 (*n* = 110)**		**CLU3 (*n* = 52)**		**CLU2 (*n* = 31)**		***p*-Value**
EE MVPA		352 ± 173		474 ± 276		555 ± 320		<0.001	484 ± 322		619 ± 347		644 ± 410		0.016
PAL		1.61 ± 0.21		1.55 ± 0.23		1.61 ± 0.21		0.051	1.58 ± 0.20		1.58 ± 0.17		1.75 ± 0.22		<0.001
Education								<0.001							0.01
	Low	34	−75.60%	15	−15.80%	22	−12.60%		21	−19.10%	14	−26.90%	5	−16.10%	
	Medium	8	−17.80%	51	−53.70%	89	−50.90%		39	−35.50%	29	−55.80%	16	−51.60%	
	High	3	−6.67%	29	−30.50%	64	−36.60%		50	−45.50%	9	−17.30%	10	−32.30%	
Household Income								<0.001							0.387
	Low	25	−55.60%	6	−6.32%	1	−0.57%		11	−10.00%	6	−11.50%	1	−3.23%	
	Mid-low	5	−11.10%	12	−12.60%	21	−12.00%		18	−16.40%	10	−19.20%	7	−22.60%	
	High	11	−24.40%	51	−53.70%	96	−54.90%		49	−44.50%	25	−48.10%	19	−61.30%	
	DK/REF	4	−8.89%	26	−27.40%	57	−32.60%		32	−29.10%	11	−21.20%	4	−12.90%	
Z-BMI-for-age		2.42 ± 0.50		2.24 ± 0.61		2.29 ± 0.55		0.208	2.3 ± 0.57		2.42 ± 0.50		1.97 ± 0.60		0.002

Data are expressed as *n* and % or mean (X) ± standard deviation. Parental education and household income are expressed as percentage and number of subjects. Statistically significant differences were calculated using chi-squared test or Kruskal–Wallis test, where appropriate. Abbreviations: EE MVPA, energy expenditure moderate and vigorous physical activity; PAL, physical activity level; Z-BMI-for-age, body mass index for age z-score.

## Supplementary Materials

The following are available online at https://www.mdpi.com/2072-6643/12/9/2536/s1, Table S1: Principal component analysis for SRS and AMS cohorts in the EsNuPi study.
