# Supplementary material for: Clustering of Dietary Patterns and Lifestyles Among Spanish Children in the EsNuPI Study
Source: Nutrients. 2020 Aug 21;12(9):2536. doi: 10.3390/nu12092536 (PMC7551863; doi:10.3390/nu12092536)
Supplement: Supplementary file 1 [file nutrients-12-02536-s001.pdf]

Table 1. Principal component analysis for SRS and AMS cohorts in the EsNuPi study.

Variables 1 to <3 years old (SRS)	Component		
	Palatable energy-dense foods	Diverse plant and animal foods	Mediterranean-like diet
Other dairy products (grams per day)	0.775		
Sugar and sweets (grams per day)	0.774	0.295	
Bakery and pastry (grams per day)	0.746		
Ready to cook (grams per day)	0.746		
Appetizers (grams per day)	0.663		
Beverages (grams per day)	0.559		0.269
Sauces and condiments (grams per day)	0.502		0.298
Oils and fats (grams per day)	0.471	0.359	0.206
Eggs (grams per day)	0.328		0.255
Cereals (grams per day)		0.797	0.354
Vegetables (grams per day)		0.758	0.274
Milk and dairy products (grams per day)		0.697	-0.341
Cereal-based baby foods (grams per day)	-0.310	0.598	-0.258
Meat and meat products (grams per day)	0.289	0.435	0.367
Nuts (grams per day)	0.219		0.704
Fruit (grams per day)		0.492	0.674
Legumes (grams per day)			0.640
Fish and shellfish (grams per day)			0.556
Variance (%)	28.017	13.359	9.891
Cumulative proportion of variance (%)	28.017	41.375	51.266

Variables 3 to <6 years old (SRS)	Component		
	Palatable energy-dense foods	Mediterranean-like diet	Component 3
Ready to cook (grams per day)	0.703	0.287	
Sugar and sweets (grams per day)	0.693		
Bakery and pastry (grams per day)	0.692		
Oils and fats (grams per day)	0.524		0.314
Other dairy products (grams per day)	0.485		
Sauces and condiments (grams per day)	0.430		
Appetizers (grams per day)	0.424		
Beverages (grams per day)	0.356		0.316
Fish and shellfish (grams per day)		0.687	
Legumes (grams per day)		0.660	

Nuts (grams per day)		0.613	-0.255
Meat and meat products (grams per day)	0.446	0.599	
Vegetables (grams per day)		0.514	0.335
Eggs (grams per day)		0.303	
Cereals (grams per day)	0.275	0.234	0.717
Cereal-based baby foods (grams per day)			0.616
Milk and dairy products (grams per day)			0.501
Fruit (grams per day)		0.462	0.494
Variance (%)	21.272	9.799	8.474
Cumulative proportion of variance (%)	21.272	31.071	39.545

Variables 6 to <10 years old (SRS)	Component		
	**Palatable energy-dense foods**	**Mediterranean-like diet**	**Component 3**
Cereals (grams per day)	0.700		
Ready to cook (grams per day)	0.664		0.259
Sauces and condiments (grams per day)	0.660		
Sugar and sweets (grams per day)	0.657		
Oils and fats (grams per day)	0.608		
Meat and meat products (grams per day)	0.535	0.395	0.348
Appetizers (grams per day)	0.455		
Eggs (grams per day)	0.407		
Vegetables (grams per day)		0.686	
Nuts (grams per day)		0.661	
Fish and shellfish (grams per day)	0.206	0.588	
Legumes (grams per day)		0.527	
Beverages (grams per day)			0.611
Milk and dairy products (grams per day)			0.560
Fruit (grams per day)		0.487	0.524
Other dairy products (grams per day)			0.524
Bakery and pastry (grams per day)	0.349		0.478
Cereal-based baby foods (grams per day)			0.329
Variance (%)	21.803	9.149	7.915
Cumulative proportion of variance (%)	21.803	30.952	38.866

Variables 1 to <3 years old (AMS)	Component		
	**Mediterranean-like diet**	**Component 2**	**Palatable energy-dense foods**
Cereals (grams per day)	0.900		
Vegetables (grams per day)	0.867		
Fruit (grams per day)	0.659		
Cereal-based baby foods (grams per day)	0.621		-0.345
Oils and fats (grams per day)	0.573		0.552
Milk and dairy products (grams per day)	0.536	0.202	-0.315
Legumes (grams per day)	0.521		0.225
Other dairy products (grams per day)		0.800	
Bakery and pastry (grams per day)		0.699	0.233
Meat and meat products (grams per day)	0.545	0.659	
Ready to cook (grams per day)		0.608	0.575
Eggs (grams per day)		0.573	
Fish and shellfish (grams per day)		0.414	0.206
Beverages (grams per day)		0.397	0.400
Appetizers (grams per day)			0.695
Sugar and sweets (grams per day)	0.229	0.277	0.633
Sauces and condiments (grams per day)		0.388	0.564
Nuts (grams per day)			
Variance (%)	28.327	15.365	6.871
Cumulative proportion of variance (%)	28.327	43.691	50.562

Variables 3 to <6 years old (AMS)	Component		
	Mediterranean-like diet	Palatable energy-dense foods	Component 3
Legumes (g/day)	0.644		0.214
Fish and shellfish (g/day)	0.636		
Vegetables (g/day)	0.559		0.556
Meat and meat products (g/day)	0.557	0.289	0.252
Nuts (g/day)	0.553		
Ready to cook/eat (g/day)	0.548	0.532	-0.227
Oils and fats (g/day)	0.3	0.244	
Sauces and condiments (g/day)		0.671	
Appetizers (g/day)	0.339	0.661	
Beverages (g/day)	-0.261	0.654	0.258
Sugars and sweets (g/day)	0.288	0.544	
Eggs (g/day)		0.442	
Other dairy products (g/day)	0.26	0.397	
Bakery and pastry (g/day)	0.249	0.375	
Fruits (g/day)			0.72
Cereals (g/day)	0.363		0.65
Milk and dairy products (g/day)			0.588
Cereal-based baby foods (grams per day)		-0.227	0.533
Variance (%)	22.611	12.115	7.573
Cumulative proportion of variance (%)	22.611	34.727	42.3

Variables 6 to <10 years old (AMS)	Component		
	**Palatable energy-dense foods**	**Mediterranean-like diet**	**Component 3**
Bakery and pastry (grams per day)	0.772		
Beverages (grams per day)	0.735	0.231	
Ready to cook (grams per day)	0.630		0.473
Oils and fats (grams per day)	0.619		
Sugar and sweets (grams per day)	0.531		0.492
Other dairy products (grams per day)	0.362		
Fruit (grams per day)		0.712	
Vegetables (grams per day)		0.706	
Cereals (grams per day)		0.616	
Milk and dairy products (grams per day)	0.238	0.485	
Legumes (grams per day)		0.245	
Sauces and condiments (grams per day)			0.765
Meat and meat products (grams per day)		0.313	0.562
Appetizers (grams per day)			0.461
Fish and shellfish (grams per day)		0.394	0.424
Nuts (grams per day)		0.322	0.379
Eggs (grams per day)			
Cereal-based baby foods (grams per day)			
Variance (%)	17.685	11.303	8.575
Cumulative proportion of variance (%)	17.685	28.989	37.564

SRS, Spanish Reference Cohort, AMS, Adapted Milk Consumers Cohort. Principal component analysis (PCA). was used to maximize the information gained for the predominant food groups from diet. This mathematical. model calculates new variables (principal components) that account for the variability in the food groups data and enables the study of covariances or correlations between variables (e.g., milk and dairy products, cereals, vegetables, meat and meat products, etc.). The combination of food groups variables with the greatest amount of variability is the first principal component. The subsequent components (second and third principal components) describe the maximum amount of remaining variability. Factor loading was used to interpret the factor structure. Loadings are equivalent to Pearson correlation coefficients, and a higher loading indicates a stronger relation between a factor and an observed variable. Strong loading was defined as a value ≥0.6, and marginal loading as a value from 0.2 to 0.4.
